# Immediate effects of Vojta Therapy on gait ability in down syndrome patients: a pilot study

**DOI:** 10.3389/fneur.2024.1511849

**Published:** 2025-01-06

**Authors:** Guoping Qian, Ewelina Perzanowska, Mirela Kozakiewicz, Paulina Ewertowska, Hongli Yu, Zbigniew Ossowski

**Affiliations:** ^1^Department of Physical Culture, Gdansk University of Physical Education and Sport, Gdansk, Poland; ^2^College of Physical Education, Sichuan University of Science & Engineering, Zigong, Sichuan, China

**Keywords:** immediate effects, spatiotemporal gait parameters, Vojta Therapy, down syndrome, Vicon, pilot study

## Abstract

**Background:**

Vojta Therapy (VT) is a neurorehabilitation approach that targets ontogenetic postural function and automatic body posture control. Research has shown its potential to enhance gait ability. However, limited evidence exists regarding its immediate effects on individuals with Down syndrome (DS).

**Objectives:**

This study aimed to assess the immediate effects of one session VT on spatiotemporal gait parameters in individuals with DS.

**Design:**

A non-randomized pilot study was conducted.

**Methods:**

Sixteen individuals with DS (mean age: 17.88 ± 4.57 years, 8 males) participated in this study. Each received a single VT session administered by an experienced physiotherapist. Spatiotemporal gait parameters before and after VT were analyzed using the Vicon motion capture system.

**Results:**

Significant improvements were observed in walking speed (m/s), cadence (steps/min), right step time (s), step length (cm), stride length (cm), and double support time (%GC) following the VT session (*P* < 0.05). These findings suggest that VT may offer immediate benefits in improving gait parameters for individuals with DS.

**Conclusions:**

Future large-scale studies with more robust designs are necessary to explore the long-term effects of extended VT programs.

## 1 Introduction

Down syndrome (DS), also known as Trisomy 21, is the most prevalent chromosomal condition associated with intellectual disability (1). As of 2015, DS prevalence in Europe was estimated to be 5.7 per 10,000 people, accounting for approximately 419,000 individuals (2). DS is linked to an increased risk of various clinical disorders, including congenital heart defects, respiratory diseases, Alzheimer's disease, gastrointestinal malformations, osteoporosis, thyroid dysfunction, epilepsy, autoimmune diseases, and disorders of the hematological and metabolic systems (1, 3–7). Moreover, DS affects the musculoskeletal system, resulting in various sequelae including muscle weakness, hypermobility and ligamentous laxity (8). These musculoskeletal issues contribute to motor coordination difficulties and alter gait patterns in individuals with DS.

Gait is widely recognized as a critical indicator of motor development, influencing cognition, social interactions, and complex motor skills such as running and jumping. Proper gait performance is essential for daily activities (9–11). However, individuals with DS often experience gait impairments characterized by reduced speed, a prolonged double-support phase, shortened step length, and increased stride width (12). These alterations in spatiotemporal gait parameters are thought to be compensatory mechanisms to enhance mechanical stability (13). Additionally, bone abnormalities, such as hip and patellar joint instability, can further impair walking ability in individuals with DS (14, 15). As a result, these gait impairments increase the energy cost of walking, limiting their ability to perform daily activities. This leads to a sedentary lifestyle, increasing dependence on others, and reduced quality of life (16–19).

The “delay hypothesis” suggests that motor development milestones in children with DS are postponed relative to typically developing children. While gait ability matures over time in typically developing children (12, 20), those with DS often maintain lower gait proficiency levels. Therefore, improving gait ability in individuals with DS is crucial to promoting health and independence throughout the lifespan. Physiotherapy has shown promise in improving gait function in DS patients. Substantial evidence supports the efficacy of therapeutic exercises, such as treadmill training and combined exercise programs, in enhancing typical gait development and independent walking ability (21, 22). These interventions promote motor milestone acquisition and increase physical activity levels. Additionally, studies have shown that a 10-week Nordic walking program for adults with DS led to significant improvements in spatiotemporal gait parameters relative to no training (23).

Vojta Therapy (VT), developed by Czech neurologist Václav Vojta, is a neuromodulative therapy based on neurophysiological principles of motor and postural control (24). This method involves applying external therapeutic stimuli to elicit automatic motor responses. It aims to modify task-related motor activation, promote typical innate patterns, and facilitate normal motor functions. VT specifically targets central nervous system (CNS) circuits that regulate automatic postural and movement adjustments (25). Unlike conventional muscle training, VT emphasizes the role of the CNS in mastering automatic movement functions, placing high demands on neural plasticity (26, 27). By integrating brain stimulation with postural awareness, VT activates both external and internal sensory receptors, which provide afferent stimulation to the CNS (25). This approach enables individuals to develop more stable and coordinated motor functions by stimulating neuroplastic responses in CNS pathways linked to movement and postural regulation. A systematic review and meta-analysis showed that VT significantly alleviated pain intensity, decreased disability severity, and improved quality of life in individuals with low back pain (28). In addition, a systematic review suggested the potential benefits of VT on respiratory function, particularly in enhancing oxygen saturation and diaphragm movement coordination in patients with neuromuscular disorders (29).

VT therapeutic effects have been objectively measured using surface electromyography and functional near-infrared spectroscopy (30). For instance, researchers observed the impact of VT on trunk-stabilizing muscles (rectus abdominis, external obliques, internal obliques, and serratus anterior) in healthy subjects using surface electromyography (31). Moreover, studies have further explained the mechanism of VT through surface electromyography, demonstrating muscle activation via spinal cord tracts, along with changes in subcortical areas (such as the putamen and cerebellum) and cortical regions, including the supplementary motor area, superior parietal cortex, premotor area, and posterior cingulate cortex (32, 33). A recent review, encompassing 55 studies, concluded that VT enhances cortical and muscle activity in adults, with clinical benefits observed in improved balance and postural control for neurological conditions like multiple sclerosis. Additionally, VT has shown efficacy in orthopedic issues, such as low back pain, by reducing pain and improving gait parameters (34). This sheds light on the neurophysiological mechanisms underlying VT therapeutic effects. VT falls within neuromodulation and pattern generators neurophysiological models, which seems to enable more effective posture control and gait improvement (35, 36).

Therefore, the primary use of VT is in treating individuals with neurogenetic disorders. There is substantial evidence indicating that VT is effective in improving postural control, spatiotemporal gait parameters, and stability in individuals with stroke and cerebral palsy (37, 38). Although the efficacy of VT in improving spatiotemporal gait parameters in persons with stroke and cerebral palsy has been well documented, to the best of our knowledge, no research has examined the immediate effects of VT on spatiotemporal gait parameters in persons with DS. To address this gap, this study explores the immediate effects of a single VT session on the spatiotemporal gait parameters of individuals with DS.

## 2 Materials and methods

### 2.1 Study design and research ethics

This pilot, non-randomized, single-group pre-post study aimed to test the immediate effects of VT on spatiotemporal gait parameters in individuals with DS. The research was approved by the Bioethics Commission of the District Medical Chamber in Gdansk (KB/23-23) and conducted in compliance with the ethical principles of the 1975 Declaration of Helsinki and its subsequent amendments. The research also complied to the Transparent Reporting of Evaluations with Non-randomized Designs (TREND) reporting guidelines (39). All participants provided written informed consent. For participants unable to provide their own consent or those who had difficulties writing, their legal guardians (typically their parents) provided written informed consent on their behalf.

### 2.2 Study population

Eligible participants were aged 10 to 30 years and met the following inclusion criteria: (1) a confirmed diagnosis of DS and (2) the ability to understand and cooperate with instructions provided by the physiotherapist during the VT session. The exclusion criteria included participants who: (1) had been enrolled in a structured physiotherapy program within 3 months prior to the study; (2) had undergone previous surgeries that might interfere with reflex creeping during VT (e.g., abdominal or head surgeries); (3) had cardiovascular conditions (e.g., congenital heart disease); (4) had neuromuscular disorders; or (5) had experienced central nervous system infections.

Recruitment began in March 2023 and was completed in April 2024. The trial was conducted at the Laboratory of Physical Effort and Genetics in Sport at the Gdansk University of Physical Education and Sport (GUPES) on May 12, 2023, and May 23, 2023.

### 2.3 Masking

The therapy was consistently administered to all participants by an experienced physiotherapist, Dr. Kozakiewicz, who was not involved in any other aspects of the study, such as data collection or analysis.

### 2.4 Intervention

A single VT session was conducted by a physiotherapist with over 20 years of clinical experience. Prior to the session, each participant underwent a face-to-face assessment. The VT session lasted approximately 20 min, during which participants completed two repetitions in a reflex creeping position for 2 min on each side (left and right), with 2–3-min rest between each position. Participants wore only shorts during the session to facilitate observation of muscle chains and ensure proper stimulation. Key elements of VT include maintaining symmetry, stabilizing support points (elbow and foot), and ensuring specifically aligned head positioning. This setup activates muscles on both sides of the body, activating motor reactions in the limbs and promoting trunk stabilization and symmetry, axial elongation. During therapy, the engagement of abdominal muscles and proper stabilization of support points activate the paraspinal muscles, aiding in spinal axial elongation (Supplementary Figure 1).

### 2.5 Outcomes

The primary outcomes were spatiotemporal gait parameters, including walking speed (m/s), cadence (steps/min), step time (s), step length (m), stride time (s), stride length (m), step width (m), double support time [as a percentage of the gait cycle (%GC)], and single support time (%GC). Data were collected in two stages: a pre-test conducted 30 min before VT and a post-test conducted within 10 min after the VT session to assess the immediate effects of therapy.

### 2.6 Data acquisition and processing

Data were collected using a 10-camera Vicon 2.0 motion capture system (VICON, Oxford Metrics Limited, UK) equipped with NIR T40S cameras, operating at 100 Hz. Data from the cameras were processed using Vicon Nexus 2.9.3 software. Vicon was chosen for its well-documented accuracy and precision in motion analysis. Previous research demonstrated that the system has a mean absolute inaccuracy of only 0.15 mm in static circumstances and below 2 mm under dynamic motion.

In accordance with the Plugin-Gait Model^1^, 16 circular reflective markers (2.5 cm in diameter) were placed on the subjects' left and right extremities limbs and spines. Marker locations comprised the anterior superior iliac spine, posterior superior iliac spine, lower third of the femur (both middle and lateral), lateral knee, lateral tibia (lower third), lateral malleolus, calcaneus, and the dorsal side of the second metatarsophalangeal joint. Participants were directed to walk barefoot at a self-selected speed on a 10-meter pathway, completing five repetitions.

### 2.7 Sample size calculation

The sample size was calculated using G-Power. We calculated the necessary sample size to ensure pilot study statistical robustness. We chose a paired *t-*test as the most appropriate statistical method, given the aim to compare pre- and post-intervention results within the same group. The calculation considered standard parameters for clinical intervention studies, including an expected medium effect size (Cohen's d = 0.50), a significance level (α) of 0.05, and a desired power (1–β) of 0.80. These parameters minimized the risks of both Type I and Type II errors, determining the smallest number of participants required to reliably detect clinically significant changes in gait characteristics. While pilot studies often have exploratory objectives and might utilize smaller sample sizes, the recommended number of participants ensures sufficient statistical power to discern meaningful trends that can inform subsequent, larger-scale research. Additionally, to accommodate potential dropouts and missing data—particularly in studies involving vulnerable populations such as individuals with DS—it is prudent to overrecruit by 10% to 15% beyond the target sample size. This approach upholds stringent scientific rigor standards, ensuring that the findings of the pilot study are both credible and reliable.

### 2.8 Data analysis

Data were analyzed with Microsoft Excel 2016 (Microsoft Corp.) and SPSS version 20.0 (IBM SPSS). Prior to analysis, the spatiotemporal gait parameters were preprocessed to remove outliers based on Grubb's test. Continuous variables were reported as mean ± standard deviation (SD) or median ± interquartile range (IQR), while categorical variables were presented as percentages (%) and sample size (N). The Shapiro-Wilk test was employed to evaluate the normality of differences between pre- and post-intervention outcomes. For normally distributed outcomes, paired sample *T*-tests were performed. Non-normally distributed outcomes were assessed using the Wilcoxon signed-rank test. Statistical significance was established at *P* < 0.05 (two-tailed).

## 3 Results

### 3.1 Study population

A total of 16 individuals with DS (*n* = 8 females; *n* = 8 males), with a mean age of 17.88 years, were recruited for this study. Table 1 illustrates baseline characteristics of all participants, with additional details available in Supplementary Table 1.

**Table 1:** Characteristics of the participants.

**Samples**	**Gender**	**Age (year)**	**Height (cm)**	**Weight (kg)**	**Body Mass Index (kg/m^2^)**
P1	F	15	168	65.10	23.07
P2	F	14	141	65.00	32.69
P3	F	12	155	49.90	20.77
P4	M	17	185	63.10	18.44
P5	M	23	160	54.60	21.33
P6	M	17	163	77.00	28.98
P7	M	18	154	42.20	17.79
P8	F	15	140	57.60	29.39
P9	M	17	156	55.20	22.68
P10	F	19	158	80.50	32.45
P11	F	23	159	69.10	27.51
P12	F	30	167	78.00	27.97
P13	M	19	164	65.80	24.46
P14	M	14	161	59.40	22.92
P15	F	13	142	48.40	24.00
P16	M	20	148	51.70	23.60

cm, centimeter; F, female; M, male; kg, kilograms; m, meter.

### 3.2 Outcomes

All participants successfully completed the VT intervention with 100% compliance and no adverse effects. The outcomes of the spatiotemporal gait parameters are described in Table 2. A Shapiro Wilk test indicated that most variables were normally distributed, including walking speed, step length (left and right), stride length (left and right), stride time (left and right) and step time (left and right). Non-normally distributed variables included cadence.

**Table 2:** Changes in spatiotemporal gait parameters after vojta therapy (*n* = 16).

**Variables**	**Pre-test (mean ±SD/median)**	**Post-test (mean ±SD/median)**	**T-value/Z-value**	**Hedge's g/R-value**
**Spatiotemporal**				
Walking speed (m/s)^**^	0.96 ± 0.26	1.12 ± 0.32	−3.85	0.91
**Spatial**				
Step length (left) (m)^*^	0.53 ± 0.10	0.58 ± 0.11	−2.74	0.81
Step length (right) (m)^**^	0.52 ± 0.11	0.57 ± 0.10	−3.89	0.92
Stride length (left) (m)^**^	1.04 ± 0.21	1.15 ± 0.20	−3.41	0.65
Stride length (right) (m)^*^	1.05 ± 0.23	1.13 ± 0.23	−2.84	0.67
**Temporal**				
Stride time (left) (s)	1.12 ± 0.12	1.08 ± 0.19	1.28	0.29
Stride time (right) (s)	1.12 ± 0.18	1.04 ± 0.16	2.13	0.51
Step time (left) (s)	0.58 ± 0.09	0.55 ± 0.12	1.23	0.30
Step time (right) (s)^**^	0.57 ± 0.08	0.53 ± 0.08	3.09	0.73
Cadence (steps/min)^*^	105.76 (101.2, 120.0)	117.00 (105.1, 127.3)	2.22	0.56
**Temporophasic**				
Single support (left) (%GC)	0.45 ± 0.09	0.42 ± 0.06	1.39	0.33
Single support (right) (%GC)	0.44 ± 0.05	0.43 ± 0.08	0.49	0.12
Double support (%GC)^*^	0.26 ± 0.05	0.23 ± 0.08	2.68	0.64

Treatment variables indicate significant differences ^**^P < 0.01 and ^*^P < 0.05. SD, standard deviation; m/s, meter/second; m, meter; s, second; %GC, % gait cycle.

### 3.3 Spatiotemporal parameters

**Figure 1:**
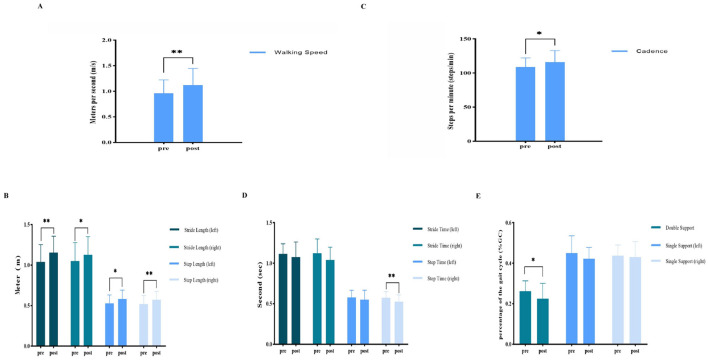
**(A)** Spatiotemporal parameters—changes in walking speed before and after the vojta therapy (VT) program. **(B)** Spatial parameters: changes in step and stride lengths (left and right) before and after the VT program. **(C)** Temporal parameters—changes in cadence before and after the VT program. **(D)** Temporal parameters—changes in step time (left and right) and stride time (left and right) before and after the VT program. **(E)** Temporophasic parameters—changes in single support (left and right) and double support before and after the VT program. ^**^Show significant changes at the 0.01 level, and ^*^show significant changes at the 0.05 level.

After a single VT session, walking speed increased significantly, from 0.96 ± 0.26 m/s to 1.12 ± 0.32 m/s (*p* < 0.01, g = 0.91). Hedge's g indicated a large effect size for this improvement (Table 2; Figure 1A).

### 3.4 Spatial parameters

The paired *t*-test revealed significant changes in all spatial parameters before and after VT. Step length (left) increased from 0.53 ± 0.10 m to 0.58 ± 0.11 m (*p* < 0.05, g = 0.81) and step length (right) increased from 0.52 ± 0.11 m to 0.57 ± 0.10 m (*p* < 0.01, g = 0.92). Stride length (left) increased from 1.04 ± 0.21 m to 1.15 ± 0.20 m (*p* < 0.05, g = 0.65) and stride length (right) increased from 1.05 ± 0.23 m to 1.13 ± 0.23 m (*p* < 0.05, g = 0.67) (Table 2; Figure 1B).

### 3.5 Temporal parameters

Significant changes were observed in two temporal parameters. Cadence increased from 105.75 (101.2, 120.0) steps/min to 117.00 (105.1, 127.3) steps/min (*p* < 0.05, g = 0.56) (Table 2; Figure 1C), and step time (right) increased from 0.57 ± 0.08 s to 0.53 ± 0.08 s (*p* < 0.01, g = 0.73). While changes were not statistically significant for stride time (left and right) and step time (left), the mean differences indicated slight changes (Table 2; Figure 1D).

### 3.6 Temporophasic parameters

Double support reduced significantly after VT, from 0.26 ± 0.05%GC to 0.23 ± 0.08%GC (*p* < 0.05, g = 0.64). Although single support (left and right) showed slight reductions, the paired *t*-tests indicated non-significant differences for both parameters (Table 2; Figure 1E).

## 4 Discussion

To the best of our knowledge, this is the first research to explore the immediate effects of VT on spatiotemporal gait parameters in individuals with DS. The results indicate that VT can significantly improve these parameters, with notable increases in gait speed, cadence, and stride length. These improvements suggest a meaningful enhancement in walking ability of DS patients following VT.

DS affects the muscles, bones, and joints, which can lead to altered gait patterns (40). Gait ability is crucial for performing daily life activities, and when impaired, it increases the dependence of individuals with DS, negatively affecting their quality of life. There is strong evidence that physiotherapy interventions focus on improving gait in people with DS (41). In individuals with DS, neuromuscular abnormalities such as hypotonia and ligamentous laxity often result in reduced gait speed and compensatory movement patterns. These compensatory strategies are evident at different stages of the gait cycle (13). A positive relationship exists between walking velocity and parameters such as cadence, step length and step time. Gait speed, often referred to as the “sixth vital sign,” has been shown in numerous studies to be linked with cognitive function, all-cause mortality, and overall health (9, 13, 42). Thus, improving gait speed is a critical goal for enhancing functional mobility of DS patients. Our study also found significant improvements in other spatiotemporal gait parameters, particularly a reduction in the percentage of double support. This decrease in double support time may indicate improved coordination following VT. Additionally, the complexity of gait was evident in the way that strengthening one parameter often led to positive changes in others.

The literature on the effectiveness of VT for improving gait in individuals with DS is scarce. However, studies in other populations have shown promising results. For instance, researchers found that an 8-week VT program improved spatiotemporal gait parameters in children with spastic diplegia (43). Similarly, researchers observed significant improvements in the 10-meter walk test (10 MWT) in individuals with multiple sclerosis following VT in a quasi-experimental controlled trial (44). There is also substantial evidence supporting the role of VT in enhancing locomotion and GMFM-88 scores in children with cerebral palsy (37, 45, 46). These findings align with the results observed in this study.

In this study, all participants completed a single VT session with two repetitions in a reflex creeping position. Each repetition lasted 2 min per side (left and right) with a 2–3-min rest between sides. In principle, this short-term VT stimulates activation of automatic, innate movement patterns that promote improved gait coordination by activating both muscular and cortical activity, particularly within the motor cortex (25). This activation may enhance postural control and motor behavior. In addition, VT has been shown to activate neuromodulate activity in the cerebellum and reticular formation through reflex creeping. This establishes essential neural circuits between the thalamus, basal ganglia, and cortex that support motor control and postural stability (47). Clinical evidence suggests that VT aligns with contemporary neuroscience principles by engaging cortical and subcortical structures, including the thalamo-cortical circuits, the basal ganglia, and the supplementary motor area—all critical for motor control and movement learning (48).

Consistent with other neurorehabilitation therapies that promote neuroplasticity, VT likely activates these circuits through sensory feedback mechanisms. This enhances neural communication within the CNS to support postural control and movement coordination (34, 49). To minimize fatigue and sustain engagement in participants with DS, we selected an intermittent VT duration of 2 min per side, maximizing the intervention's effectiveness. A recent pilot study on individuals with chronic stroke aligns with our findings. This study demonstrated that a single VT session significantly improved walking speed and balance. This supports the immediate effects of VT on improving gait ability across different populations (50). In conclusion, this CNS-targeted activation reinforces stable gait patterns and facilitates motor learning, which may explain the observed improvements in spatiotemporal gait parameters in individuals with DS.

### 4.1 Limitations and strengths

This pilot study represents a pioneering effort to evaluate the acute effects of VT on spatiotemporal gait parameters in individuals with DS. The strengths of this study include high adherence rates and strict compliance with the TREND statement. In addition, the assessor was independent of the person doing the treatment, improving the objectivity of the measurements. Additionally, the study provides preliminary evidence supporting the immediate effectiveness of a single VT session in improving gait parameters in individuals with DS.

However, several limitations should be considered. First, the study did not include a control group, which may have introduced potential bias, such as the Hawthorne effect. This limitation is inherent to the pilot nature of the study, and the results should be interpreted cautiously. Second, the limited sample size may limit the statistical power of the findings. This makes it challenging to generalize the results to broader populations or other settings, such as public nursing homes. Lastly, no long-term follow-up was conducted. Although long-term efficacy is essential for evaluating neuromodulation techniques, this study intentionally focused on the acute effects of VT. Future research is recommended to explore the mid- and long-term outcomes of VT to better understand its sustained impact on gait ability. Therefore, larger, multicenter, randomized, double-blind, and controlled clinical trials are necessary to further validate these findings and explore the long-term effects of VT.

## 5 Conclusions

This pilot study demonstrates that VT can have an immediate positive impact on spatiotemporal gait parameters, including cadence, gait speed, and double support, in individuals with DS. These results provide valuable insights for physiotherapists and clinicians. They suggest that VT could be a beneficial addition to treatment strategies aimed at improving gait ability in individuals with DS. However, the findings should be interpreted cautiously, considering the study's limitations. Further research with larger sample sizes and long-term follow-up is necessary to confirm and expand upon these preliminary results.

## Data Availability

The original contributions presented in the study are included in the article/Supplementary material, further inquiries can be directed to the corresponding author.

## Footnotes

^1^ https://help.vicon.com/space/Nexus216/11607059/Plug-in+Gait+Reference+Guide

## Supplementary material

Supplementary Table 1 Characteristics of the participants (*n* = 16).

Supplementary Figure 1 Reflex creeping performed in Vojta therapy.
