# Corrigendum: Immediate effects of Vojta Therapy on gait ability in down syndrome patients: a pilot study

**DOI:** 10.3389/fneur.2025.1591573

**Published:** 2025-04-02

**Authors:** Guoping Qian, Ewelina Perzanowska, Mirela Kozakiewicz, Paulina Ewertowska, Hongli Yu, Zbigniew Ossowski

**Affiliations:** ^1^Department of Physical Culture, Gdansk University of Physical Education and Sport, Gdansk, Poland; ^2^College of Physical Education, Sichuan University of Science & Engineering, Zigong, Sichuan, China

**Keywords:** immediate effects, spatiotemporal gait parameters, Vojta Therapy, down syndrome, Vicon, pilot study

In the published article, there were errors in [Table 1, Table 2, and Figure 1] as published. [For Table 1: While the heading “Height (cm)” is correct, the actual values were incorrectly recorded in meters. For Table 2: Although the data (Spatial) were presented in meter (m), the original labels were incorrectly marked as centimeter (cm); additionally, the original title “Table 2. Changes in spatiotemporal gait parameters after home-based vojta therapy (*n* = 16)” was incorrect and should be corrected to “Table 2. Changes in spatiotemporal gait parameters after vojta therapy (*n* = 16). For Figure 1: the legend for Figure 1 (B) was incorrectly labeled as “centimeter (cm)” instead of “meter (m)”; additionally, the original figure title “Figure 1. **(A)** Spatiotemporal parameters—changes in walking speed before and after the home-based vojta therapy (VT) program. **(B)** Spatial parameters: changes in step and stride lengths (left and right) before and after the home-based VT program. **(C)** Temporal parameters—changes in cadence before and after the home-based VT program. **(D)** Temporal parameters—changes in step time (left and right) and stride time (left and right) before and after the home-based VT program. **(E)** Temporophasic parameters—changes in single support (left and right) and double support before and after the home-based VT program.” should be corrected to “Figure 1. **(A)** Spatiotemporal parameters—changes in walking speed before and after the vojta therapy (VT) program. **(B)** Spatial parameters: changes in step and stride lengths (left and right) before and after the VT program. **(C)** Temporal parameters—changes in cadence before and after the VT program. **(D)** Temporal parameters—changes in step time (left and right) and stride time (left and right) before and after the VT program. **(E)** Temporophasic parameters—changes in single support (left and right) and double support before and after the VT program.”]. The corrected [Table 1; Table 2 and Figure 1] and its caption [Table 1. Characteristics of the participants; Table 2. Changes in spatiotemporal gait parameters after vojta therapy (*n* = 16) and Figure 1. **(A)** Spatiotemporal parameters—changes in walking speed before and after the vojta therapy (VT) program. **(B)** Spatial parameters: changes in step and stride lengths (left and right) before and after the VT program. **(C)** Temporal parameters—changes in cadence before and after the VT program. **(D)** Temporal parameters—changes in step time (left and right) and stride time (left and right) before and after the VT program. **(E)** Temporophasic parameters—changes in single support (left and right) and double support before and after the VT program.] appear below.

**Table 1:** Characteristics of the participants.

**Samples**	**Gender**	**Age (year)**	**Height (cm)**	**Weight (kg)**	**Body Mass Index (kg/m^2^)**
P1	F	15	168	65.10	23.07
P2	F	14	141	65.00	32.69
P3	F	12	155	49.90	20.77
P4	M	17	185	63.10	18.44
P5	M	23	160	54.60	21.33
P6	M	17	163	77.00	28.98
P7	M	18	154	42.20	17.79
P8	F	15	140	57.60	29.39
P9	M	17	156	55.20	22.68
P10	F	19	158	80.50	32.45
P11	F	23	159	69.10	27.51
P12	F	30	167	78.00	27.97
P13	M	19	164	65.80	24.46
P14	M	14	161	59.40	22.92
P15	F	13	142	48.40	24.00
P16	M	20	148	51.70	23.60

cm, centimeter; F, female; M, male; kg, kilograms; m, meter.

**Table 2:** Changes in spatiotemporal gait parameters after vojta therapy (*n* = 16).

**Variables**	**Pre-test (mean ±SD/median)**	**Post-test (mean ±SD/median)**	**T-value/Z-value**	**Hedge's g/R-value**
**Spatiotemporal**				
Walking speed (m/s)^**^	0.96 ± 0.26	1.12 ± 0.32	−3.85	0.91
**Spatial**				
Step length (left) (m)^*^	0.53 ± 0.10	0.58 ± 0.11	−2.74	0.81
Step length (right) (m)^**^	0.52 ± 0.11	0.57 ± 0.10	−3.89	0.92
Stride length (left) (m)^**^	1.04 ± 0.21	1.15 ± 0.20	−3.41	0.65
Stride length (right) (m)^*^	1.05 ± 0.23	1.13 ± 0.23	−2.84	0.67
**Temporal**				
Stride time (left) (s)	1.12 ± 0.12	1.08 ± 0.19	1.28	0.29
Stride time (right) (s)	1.12 ± 0.18	1.04 ± 0.16	2.13	0.51
Step time (left) (s)	0.58 ± 0.09	0.55 ± 0.12	1.23	0.30
Step time (right) (s)^**^	0.57 ± 0.08	0.53 ± 0.08	3.09	0.73
Cadence (steps/min)^*^	105.76 (101.2, 120.0)	117.00 (105.1, 127.3)	2.22	0.56
**Temporophasic**				
Single support (left) (%GC)	0.45 ± 0.09	0.42 ± 0.06	1.39	0.33
Single support (right) (%GC)	0.44 ± 0.05	0.43 ± 0.08	0.49	0.12
Double support (%GC)^*^	0.26 ± 0.05	0.23 ± 0.08	2.68	0.64

Treatment variables indicate significant differences ^**^P < 0.01 and ^*^P < 0.05. SD, standard deviation; m/s, meter/second; m, meter; s, second; %GC, % gait cycle.

**Figure 1:**
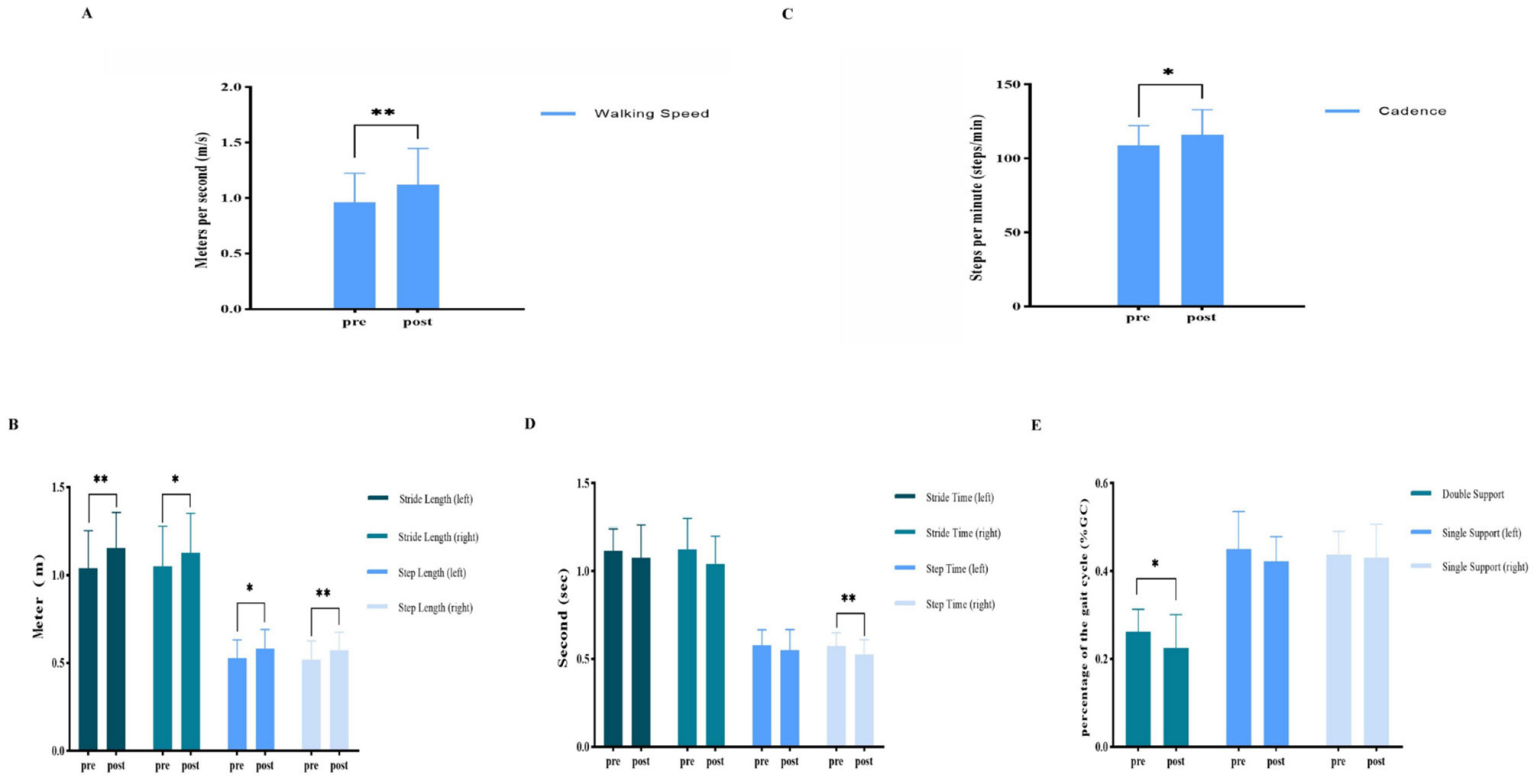
**(A)** Spatiotemporal parameters—changes in walking speed before and after the vojta therapy (VT) program. **(B)** Spatial parameters: changes in step and stride lengths (left and right) before and after the VT program. **(C)** Temporal parameters—changes in cadence before and after the VT program. **(D)** Temporal parameters—changes in step time (left and right) and stride time (left and right) before and after the VT program. **(E)** Temporophasic parameters—changes in single support (left and right) and double support before and after the VT program. ^**^Show significant changes at the 0.01 level, and ^*^show significant changes at the 0.05 level.

There was also an error in the legend for Table 2 as published. The abbreviation was incorrectly listed as “cm: centimeter” instead of “m: meter.” The corrected legend appears below.

In the published article, there was an error in the name of the ethics review institution. This correction pertains only to the name of the ethics review institution and does not affect the ethical approval itself, the study procedures, or the validity of the research findings.

A correction has been made to the section **Materials and methods**, *2.1 Study design and research ethics*. This sentence previously stated:

“The research was approved by the Ethics Review Committee of the Gdansk University of Medical Sciences (KB/23-23) and conducted in compliance with the ethical principles of the 1975 Declaration of Helsinki and its subsequent amendments.”

The corrected sentence appears below:

“The research was approved by the Bioethics Commission of the District Medical Chamber in Gdansk (KB/23-23) and conducted in compliance with the ethical principles of the 1975 Declaration of Helsinki and its subsequent amendments.”

In the published article, there was an error. The classification of normally and non-normally distributed variables. This correction does not impact the analysis or the conclusions drawn from the data.

A correction has been made to **Results**, *3.2 Outcomes*. This sentence previously stated:

“A Shapiro Wilk test indicated that most variables were normally distributed, including walking speed, cadence, step length (left and right), stride time (left and right), stride length (left and right), and step time (left). Non-normally distributed variables included step time (right) and single support (right).”

The corrected sentence appears below:

“A Shapiro Wilk test indicated that most variables were normally distributed, including walking speed, step length (left and right), stride length (left and right), stride time (left and right) and step time (left and right). Non-normally distributed variables included cadence.”

In the published article, there was an error. The units for step length and stride length were incorrectly labeled as centimeter (cm) instead of meter (m). This correction only involves unit labeling and does not alter the data analysis, results, or conclusions.

A correction has been made to **Results**, *3.4 Spatial parameters*. This sentence previously stated:

“The paired *t*-test revealed significant changes in all spatial parameters before and after VT. Step length (left) increased from 0.53 ± 0.10 cm to 0.58 ± 0.11 cm (*p* < 0.05, g = 0.81), and step length (right) increased from 0.52 ± 0.11 cm to 0.57 ± 0.10 cm (*p* < 0.01, g = 0.92). Stride length (left) increased from 1.04 ± 0.21 cm to 1.15 ± 0.20 cm (*p* < 0.05, g = 0.65), and stride length (right) increased from 1.05 ± 0.23 cm to 1.13 ± 0.23 cm (*p* < 0.05, g = 0.67) (Table 2; Figure 1B).”

The corrected sentence appears below:

“The paired *t*-test revealed significant changes in all spatial parameters before and after VT. Step length (left) increased from 0.53 ± 0.10 m to 0.58 ± 0.11 m (*p* < 0.05, g = 0.81) and step length (right) increased from 0.52 ± 0.11 m to 0.57 ± 0.10 m (*p* < 0.01, g = 0.92). Stride length (left) increased from 1.04 ± 0.21 m to 1.15 ± 0.20 m (*p* < 0.05, g = 0.65) and stride length (right) increased from 1.05 ± 0.23 m to 1.13 ± 0.23 m (*p* < 0.05, g = 0.67) (Table 2; Figure 1B).”

The authors apologize for these errors and state that this does not change the scientific conclusions of the article in any way. The original article has been updated.

