# Supplementary material for: Immediate effects of Vojta Therapy on gait ability in down syndrome patients: a pilot study
Source: Front Neurol. 2025 Jan 6;15:1511849. doi: 10.3389/fneur.2024.1511849 (PMC11743272; doi:10.3389/fneur.2024.1511849)
Supplement: Supplementary Table 1 — Characteristics of the participants (n = 16). [file Table_1.docx]

Supplementary Material

**Supplementary Table 1.** Characteristics of the participants (n = 16).

| Gender (F/M) | 8/8 |
| --- | --- |
| Age (year) | 17.88 ± 4.57 |
| Height (cm) | 157.5 ± 11.5 |
| Weight (kg) | 61.41 ± 11.13 |
| BMI (kg/m^2^) | 24.88 ± 4.53 |
| Ethnicity (white) | 16 (100%) |

Note: Values are presented as a number or mean ± standard deviation (S.D.). The abbreviations used in the table stand are as follows: BMI: body mass index; cm: centimeter; F: female; M: male; kg: kilograms.
