# Supplementary figures and images for: Immediate effects of Vojta Therapy on gait ability in down syndrome patients: a pilot study
Source: Front Neurol. 2025 Jan 6;15:1511849. doi: 10.3389/fneur.2024.1511849 (PMC11743272; doi:10.3389/fneur.2024.1511849)

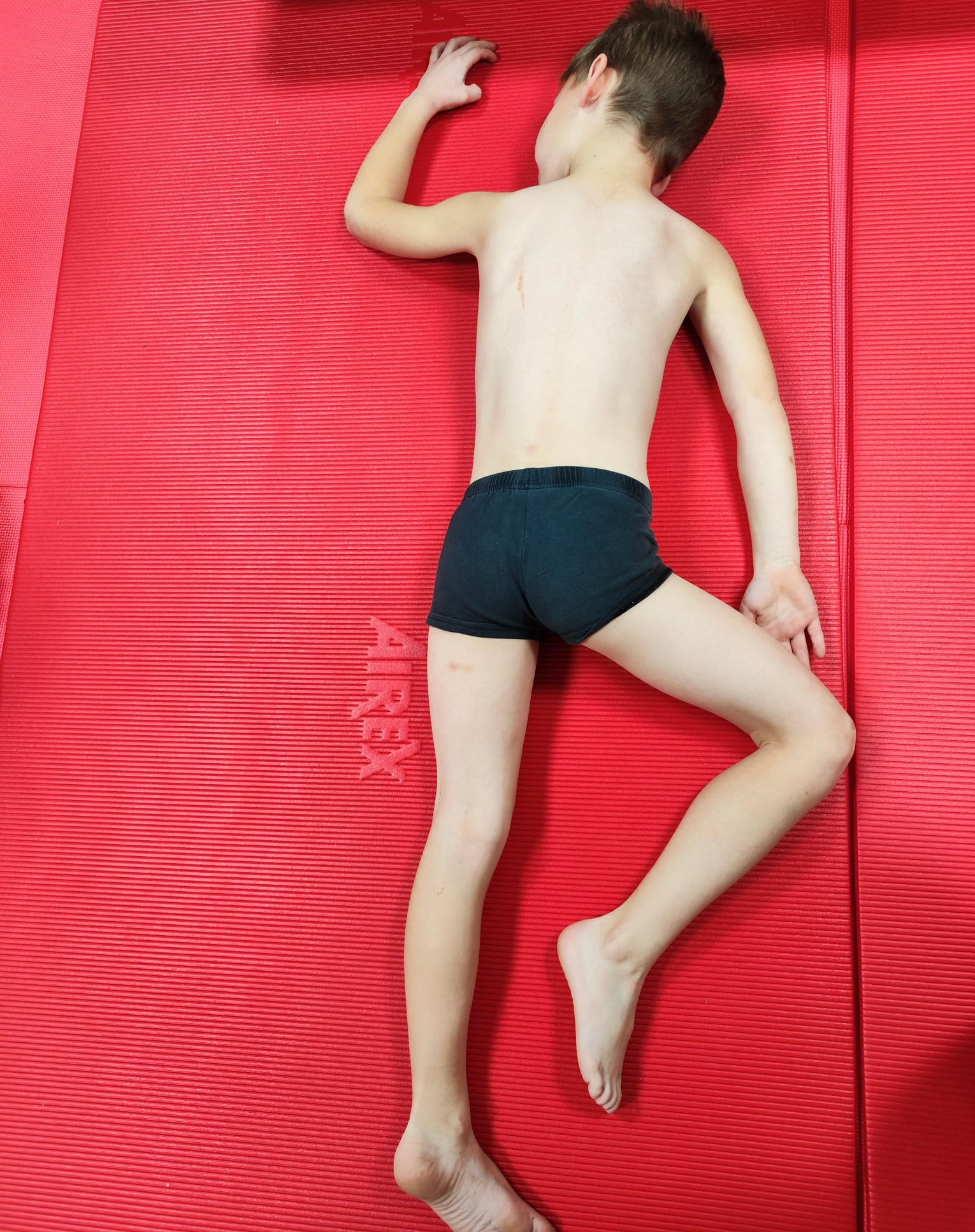

Supplement: Supplementary Figure 1 — Reflex creeping performed in Vojta therapy. [file Image_1.tif]
